# Ground glass pulmonary nodules: their significance in oncology patients and the role of computer tomography and 18F–fluorodeoxyglucose positron emission tomography

**DOI:** 10.1186/s41824-017-0021-z

**Published:** 2018-02-26

**Authors:** Laura Evangelista, Annalori Panunzio, Elena Scagliori, Paolo Sartori

**Affiliations:** 1 Nuclear Medicine Unit, Veneto Institute of Oncology IOV – IRCSS, Via Gattamelata, 64 35128 Padua, Italy; 2 Radiology Service, Ospedale Civile SS. Giovanni e Paolo, Venice, Italy; 3 Department of Radiology, Hospital San Donà di Piave, Venice, Italy

**Keywords:** Pulmonary nodules, Ct, Pet/Ct

## Abstract

**Objective:**

to determine the clinical significance of ground glass pulmonary nodules, either pure (GGNs) or mixed with the presence of solid component (MPNs), in patients with known pulmonary or extra-thoracic malignancies and to evaluate the role of computed tomography (CT) and positron emission tomography (PET)/CT in their diagnosis and follow-up.

**Methods:**

A total of 130 nodules in 68 patients were revealed: 119 GGNs and 11 MPNs. GGN lesions were found in 58 patients, MPNs in eight, and in two cases, both. The median diameter of the nodules was 7 mm (3–30 mm). Moreover, 27 patients, who had a pars-solid >5 mm in the GGN or a pure GGN with a diameter > 5 mm, underwent FDG PET/CT. The median follow-up with CT was >3 years.

**Results:**

The comparison between the first and the last positive CT scan showed that GGNs and/or MPNs remained unchanged for a median period of 18 months (range 11–48 months) in 53 patients, they disappeared after a median of 3.5 months (range 2–11 months) in 12 and increased in diameter after a median period of 17 months (range 12–67 months) in 3. In particular of these latter patients, two had malignant lesions. Only three patients with a single nodule showed a significant uptake of FDG at PET/CT.

**Conclusion:**

in the evaluation of GGNs and MNPs, CT examinations performed after 3 months often showed some changes, mainly with respect to nodules disappearing. PET/CT often plays no role but it can exclude malignancy at the end of staging. Finally, in patients with known pulmonary or extra-thoracic malignancies showing GGNs or MPNs, a 3-year CT follow up is justified, due to the slow growth rate of these lesions.

## Introduction

With the advent of multidetector CT scanners (MDCT), the presence of pulmonary nodules (PN) consisting of solid tissue of undetermined significance and with a diameter less than or equal to 3 cm, is becoming relatively frequent, in both healthy patients (undergoing CT to screen for lung cancer) and patients who undergo CT for the staging and the follow-up of thoracic cancer and that of other organs or systems. The morphological aspect of the SPN can be variable. The pure ground glass (GGNs) and part-solid nodules or part-solid GGNs are constituted of a non-solid part and a concomitant solid and non-solid part, respectively. The pathological significance of GGNs or part-solid GGNs can be that of a benign lesion (i.e. inflammation or fibrosis), a premalignant lesion (i.e. atypical adenomatoid hyperplasia), or a malignant lesion (i.e. pulmonary adenocarcinoma) (JY et al., 2007). GGNs are usually small in size, with a diameter between 5 and 10 mm, and therefore, almost undetectable with the standard radiological examination. Consequently, follow-up must necessarily be done with CT examination.

The clinical evaluation and follow-up of GGNs and part-solid GGNs derives from the results of lung cancer screening studies in groups of patients matched for age and exposure to cigarette smoke, without comorbidities (Henschke et al., 2002; Austin, 2011; Hasegawa et al., 2000). The clinical significance of GGNs and part-solid GGNs identified by MDCT in patients with a history of cancer has scarcely been investigated, although some studies have reported sporadic cases of lung metastasis as GGNs in patients with gastric cancer, lung adenocarcinoma and endometrial sarcoma (Gaeta et al., 1996). However, it cannot be excluded that a patient with known pulmonary and extra-pulmonary malignancy may develop another primary lung cancer. Recent studies have tried to assess the usefulness of 18F–Fluorodeoxyglucose (FDG) positron emission tomography (PET) in the differentiation between malignant vs. benign GGNs (Chun et al., 2009; Kim et al., 2012a; Chiu et al., 2012).

Therefore, the objectives of this study are to: 1) determine the clinical significance of GGNs, either pure or with a part solid component, in patients with known pulmonary and extra-thoracic malignancies and 2) evaluate the role of CT and PET/CT in their diagnosis and follow-up.

## Methods

### Patient eligibility.

**Fig. 1:**
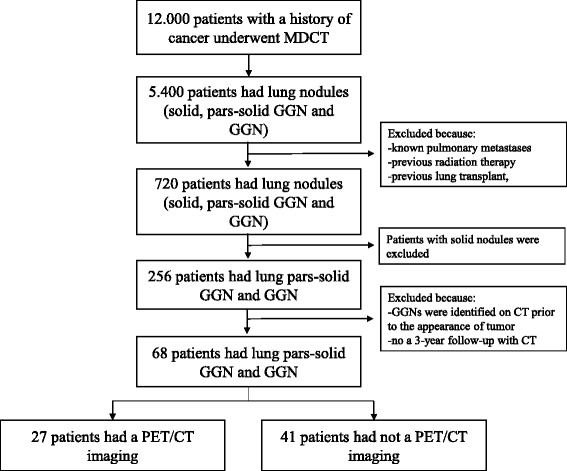
Flow-chart for the selection of study population

From April 2008 to June 2012, about 12,000 patients with a history of malignancy were sent to the service of Radiology Oncology Unit of Veneto Institute of Oncology IOV – IRCCS in Padua, for the staging or follow-up of their disease. All CT examinations were reviewed to evaluate the presence of GGNs and/or part-solid GGNs. Patients were excluded in the cases of pulmonary metastases, radiation therapy, a previous lung transplant, and, in the case of GGNs, if they were identified on CT prior to the appearance of a tumor that was considered to be a benign lesion. GGNs were considered benign based on the stability of their size and density at CT imaging, and on the absence of a solid component over at least 3 years (Godoy & Naidich, 2012). Out of 12,000 patients, 68 subjects showed GGNs and/or part-solid GGNs at CT and were included in the study (0.60%) (Fig. 1).

Subjects were 30 males and 38 females with a median age of 63.4 years (range 34–86 years). Seventeen patients had a history of breast cancer, 14 colorectal cancer, eight esophageal-gastric cancer, five cutaneous melanoma, four lung cancer, four soft tissue sarcoma, three testicular cancer, three kidney cancer, three pancreatic cancer, two ovarian cancer, one bladder cancer, one laryngeal cancer, one thyroid cancer, one prostate cancer, and one intestinal lymphoma. All patients were treated with surgery for primary tumor, with or without chemotherapy (neoadjuvant and/or adjuvant). Ten patients were treated with chemotherapy at the time of CT, nine of these (90%) were treated with potentially pneumotoxic agents (sorafenib, sunitinib, taxol, cisplatin). Twenty-five patients had secondary lesions in other distant organs. In particular, 13 patients had visceral lesions (five had solid pulmonary nodules, seven had hepatic lesions, and one had pancreatic lesion). Moreover, metastases from primary tumor, such as lymph node localization, bone and peritoneal lesions were observed in 4 cases, in 1 and in 2, respectively. Finally, lesions localized both in visceral and non-visceral (lymph node and bone) sites were observed in 5 cases.

Initial staging of the tumor was assessed according to the VII Classification of the American Joint Committee on Cancer (AJCC) and the Working Formulation Classification for Lymphoma. In particular, 10 patients were stage I, 11 were stage II, 14 were stage III, and 20 were stage IV. In 13 patients, the initial stage was unknown. Out of 68 enrolled patients, 27 subjects with a pars-solid >5 mm in the GGN or a pure GGN with a diameter > 5 mm underwent PET/CT whole-body scan within 3 months of CT.

Eleven subjects had a known history of inflammatory lung disease (one with tuberculosis and nine with lobar pneumonia). All male patients and 8/38 (21%) female patients were cigarette smokers at the time of primary tumor diagnosis (Table 1). None of the patients had clinical signs or symptoms, or laboratory findings of systemic illness at the time of CT examination.

**Table 1:** Patient characteristics

*n*	*68*
Age, years (median; range)	63.4 (34–86)
Sex, n (%)	
Male	30 (44)
Female	38 (56)
Pathology, n (%)	
Breast	17 (25)
Colon-rectal	14 (21)
Esophageal-gastric	8 (12)
Melanoma	5 (7)
Lung	4 (6)
Soft tissue sarcoma	4 (6)
Testicle	3 (5)
Kidney	3 (5)
Pancreas	3 (5)
Ovary	2 (3)
Bladder	1 (1)
Larynx	1 (1)
Thyroid	1 (1)
Prostate	1 (1)
Intestinal lymphoma	1 (1)
Stage of disease, n (%)	
Stage I	10 (15)
Stage II	11 (16)
Stage III	14 (21)
Stage IV	20 (29)
NA	13 (19)
Smoking	
No	30 (44%)
Yes	38 (56%)
History of lung pathology, n (%)	
No	58 (85)
Yes	10 (15)

### CT imaging.

All patients underwent CT of the chest, abdomen, and pelvis to identify metastases. All chest CT examinations were acquired from the lung apices through the lung bases using 8-row detector CT (Eclos; Hitachi Medical Corporation, Tokyo, Japan) or 40-row detector CT (Somatom Definition AS; Siemens Medical Systems, Erlangen, Germany) using the following parameters: section thickness of 2.5 and 3 mm, reconstruction (Bf70 algorithm) 2.5 or 2 mm, gantry rotation time 0.8 or 0.5 s, pitch 0.7 or 0.8, tube potential 120 kV, and mAs setting adjusted for body weight. All patients received intravenous contrast medium (2 mL/kg; flow rate 3 mL/s; Omnipaque 350; GE Healthcare-Milano-Italy).

CT images were reviewed and interpreted by two radiologists (S.E and P.R.) with 3 and 5 years experience of chest CT interpretation. Both specialists were presented with the patients’ clinical history, but were unaware of histological findings. Pulmonary nodules were assessed for size, number, location (upper, middle or lower lobe). Nodule size was defined as the largest diameter measured with electronic calipers on the CT images. Moreover, the nodules were classified according to their size, attenuation (solid or partly solid or ground glass) and growth.

### PET/CT protocol.

Whole body 18F–FDG PET/CT was performed using a dedicated PET/CT scanner (Biograph 16 HT, by Siemens Medical Solutions, Illinois, U.S.A.). The PET component is a high-resolution scanner with a spatial resolution of 4.7 mm and has no septa, thus allowing 3-dimensional–only acquisitions. The CT portion of the scanner is the Somatom Sensation 16-slice CT. Together with the PET system, the CT scanner is used for both attenuation correction of PET data and localization of 18F–FDG uptake in PET images. All patients were advised to fast for at least 6 h before the integrated PET/CT examination. After injection of 3.0 MBq of 18F–FDG per kg/body weight, patients rested for a period of about 60 min in a comfortable chair. A low dose CT was performed for the attenuation correction. Emission images ranging from the proximal femur and the base of the skull were acquired for 3 min per bed position. Acquired images were reconstructed using the attenuation weighted-OSEM (ordered subset expectation maximization) iterative reconstruction, with 2 iterations, 8 subsets. Fourier rebinning was used to reduce the 3D dataset to a 2-dimensional equivalent dataset, and a 4-mm full width at half maximum Gaussian filter was applied to the image after reconstruction along the axial and transaxial directions. The data were reconstructed over a 128 × 128 matrix with 2-mm pixel size and slice thickness. Processed images were displayed in coronal, transverse, and sagittal planes. Two experienced nuclear medicine physicians interpreted the FDG PET/CT images unaware of previous study findings. At visual analysis, increased FDG uptake not corresponding to physiological uptake patterns or in any foci of increased uptake corresponding to a CT abnormality (tissue and/or lymph node) were recorded as positive. On the contrary, the absence of uptake was used to define a negative PET/CT finding.

The maximum standardized uptake value (SUVmax) was determined by drawing iso-volumetric regions of interest (VOI) on the attenuation corrected FDG PET/CT images around suspected lesions. In case of multiple metastases, the lesion with the most intense uptake were analyzed.

### Statistical analysis

Continuous variables were expressed as median (maximum and minimum value: range) while categorical data as percentages (%).

## Results

In all 68 selected patients, the GGNs and part-solid GGNs were observed after a median of 15.68 months (range: 1–164 months) from the diagnosis of the primary tumor. In particular, 41 had a single lesion and 27 had multiple lesions (60.3 and 39.7%, respectively).

Moreover, 58 patients (85.3%) had GGNs: 34 had a single lesion (58.6%) and 24 had multiple lesions (41.4%).

Part-solid GGNs were present in the remaining 10 patients (14.7%): seven had only a single part-solid GGN, two had multiple GGNs with a single part-solid GGN and one had two part-solid GGNs without associated GGNs. A total of 130 nodules were found in all 68 patients. The number of nodules in each patient with multiple nodules ranged from 2 to 15. In total, there were 119 GGNs (91.5%) and 11 part-solid GGNs (8.4%). The total diameter of these nodules ranged from 5 to 30 mm (median 7.0 mm). In 10 patients with part-solid GGNs, the diameter of the solid component ranged from 3 to 22 mm (median 6 mm).

None of these nodules showed pseudo-excavations, air bronchogram, retraction of the pleura, or polycyclic margins. All nodules with ground-glass component demonstrated blurred contours.

The distribution of multiple lesions was unilateral in 7 patients (25.9%), and bilateral in 20 (74.1%). The nodules were localized accordingly: 49 in the upper lobe, 29 in the lower lobe, 10 in the middle lobe, and 6 in the lingular lobe (Table 2).

**Table 2:** Characteristics of pulmonary nodules (patient-based analysis)

Nodule number, *n (%)*	
Single	41 (60.3)
Multiple (from 2 to 15)	27 (39.7)
Nodule density, *n (%)*	
GGNs	58 (85.3)
MPN	8 (11.8)
MPN + GGNs	2 (2.9)
Diameter (mm) of ground glass component (median; range)	7 (5–30)
Diameter (mm) of solid component (median; range)	6 (3–22)
Distribution of multiple nodules, *n (%)*	
Monolateral	7 (25.9)
Bilateral	20 (74.1)
Location, *n (%)*	
Upper lobe	49
Middle lobe	10
Lower lobe	29
Lingular	6
PET/CT, *n (%)*	27
Negative	25 (93)
Positive	2 (7)

*GGN* ground glass nodule, *MPN* mixed pulmonary nodule

From the comparison between the first and the last positive CT scans, GGNs and/or part-solid GGNs (78%) remained unchanged for a median period of 18 months (range 11–48 months) in 53 patients, disappeared after a median of 3.5 months (range 2–11 months) in 12 (18%) and increased in diameter after a median period of 17 months (range 12–67 months) in 3 (4%).

**Fig. 2:**
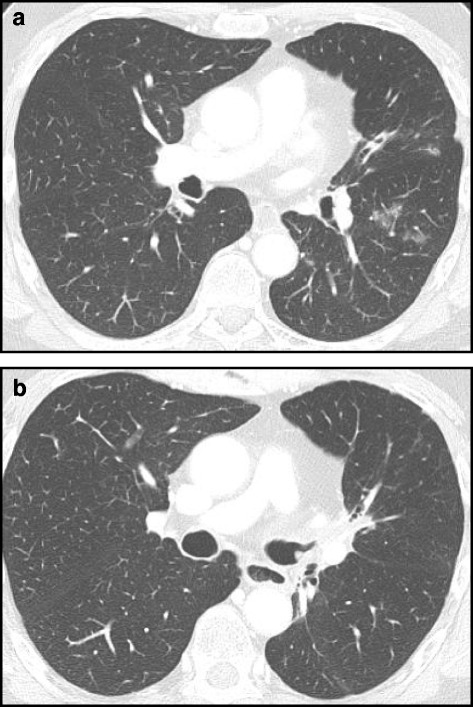
(**a**) CT scan of a patient in treatment with Sorafenib showed, in the left lung, multiple ground glass nodules in the posterior segment of superior lobe. (**b**) CT image in the same patient after 4-week withdrawal from chemotherapeutic agent, demonstrating the disappearance of the nodules

Among 53 patients showing unchanged nodules, 30 (56.6%) had a single GGN, 16 (30.2%) had multiple GGNs, six (11.3%) had a single part-solid GGN (a solid component with a diameter ranged between 3 and 6 mm), one (1.9%) had a part-solid GGN (with a solid portion of 4 mm in diameter) associated with multiple part-solid GGNs. In 12 patients with cleared lesions, all of the lesions were GGNs. In this subset of patients, four (33.3%) had a single GGN and eight (66.6%) had multiple GGNs. Of these latter eight patients, four had been taking pneumotoxic drugs (sorafenib, suitinab, cisplatin) but all subjects withdrew from them when pulmonary nodules were discovered at CT imaging (Fig. 2 a,b).

**Fig. 3:**
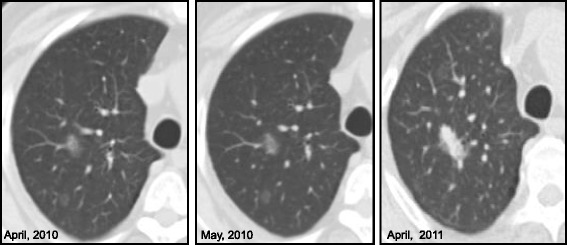
A 69-year old man with colon-rectal cancer already treated in September 2008. In April 2010, CT scan showed, in right lung, two ground glass opacities in posterior segment of upper lobe. In May 2010, the nodules were stable on CT imaging. After 12-months (April, 2011), one ground glass opacity disappeared, while the other became a solid nodule. The final histological diagnosis was metastasis from lung adenocarcinoma

Out of three cases with nodules increasing in diameter, one had a single part-solid GGN with a solid component of about 3 mm in diameter and a total nodule diameter of 8 mm. This patient had a history of colon-rectal cancer. During the follow-up at 14 months, the nodule had become completely solid (total diameter of 12 mm) and the patient (in complete remission for the first tumor) was subjected to surgical treatment, with a final histological diagnosis of lung adenocarcinoma (Fig. 3). In the other case, the patient had two bilateral part-solid GGNs, a previous history of pulmonary adenocarcinoma associated with liver metastases. The nodules grew very slowly, in fact the initial diameters were 5 and 3 mm growing to 7 and 4 mm, respectively, after 14 months, considering both the total diameter and the solid component. These nodules were considered to be metastases, after confirmation via cytological exam.

The last patient showed a part-solid GGN in combination with multiple GGNs. He had a previous history of prostate cancer in hormonal therapy. An constant increase in diameter of the nodules was observed until the last control performed 67 months from the first appearance (from 30 × 14 to 40x32mm). No metastases were found in other organs and the patient did not undergo PET/CT or biopsy of the lesions.

In all cases, the ground glass component and the solid component had increased in size (median increase: 12 mm and 16 mm for ground glass and solid components, respectively).

In patients with known lung metastases (*n* = 5), GGNs and part-solid GGNs remained unchanged (three cases of multiple GGNs, one with single part-solid GGN) or disappeared (one case with GGN multiple).

In none of the 11 patients who underwent a CT exam within one month of the first scan, had the nodules disappeared or changed.

### PET/CT analysis.

Only patients with a pars-solid >5 mm in the GGN or a pure GGN with a diameter > 5 mm underwent PET/CT (*n* = 27; 40%). Only three out of 27 patients showed a significant uptake of metabolic tracer in the lung nodules, reporting a SUV max of 5.91, 6.20 and 12.72, respectively. In all three cases, only a single lesion in the lung was observed. Each lesion had a solid component, which increased in two and remained unchanged in one of the cases as compared to the previous CT scan. The diameters of the all lung nodules with a significant FDG uptake were 12, 8 and 15 mm, respectively. The solid component of GGN showed a diameter > 5 mm.

In the first and the third patient, the lesions showed a significant uptake of FDG at PET/CT image, which was consistent with the histological diagnosis of pulmonary adenocarcinoma.

In the second case, the patient was considered as affected by a metastatic lung adenocarcinoma, associated with distant organ involvement (liver and lymph nodes).

On the contrary, in the remaining 24 cases, PET/CT resulted negative for pulmonary involvement (all GGNs or part-solid GGNs had a solid component diameter < 8 mm), except in 10 subjects, local or distant metastases was demonstrated.

**Fig. 4:**
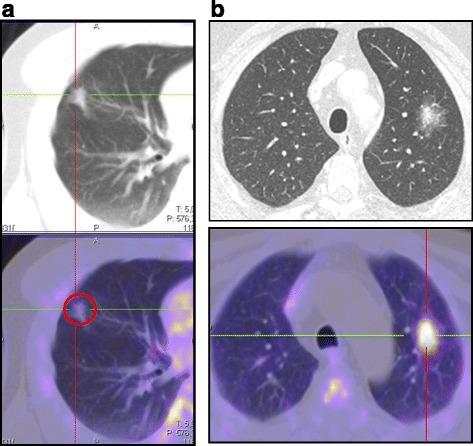
Examples of PET/CT imaging. (**a**) A 75 year-old man with a previous history of colon-rectal cancer. A CT imaging showed a sub-solid lung nodule (diameter of pars-solid was 9 mm). FDG PET/CT was negative. After 18 months of follow-up, the nodules disappeared. (**b**) A 72 year-old man with a previous prostate cancer treated with hormonal therapy from 2007. In 2010, CT imaging revealed a sub-solid lung nodule. PET/CT demonstrated a significant uptake of FDG in the solid component of the nodule. The histopathological analysis was definitive for a lung adenocarcinoma

A patient with breast cancer and a single part-solid GGN (unchanged at follow-up) was subjected to bronchoscopic examination, which showed the presence of a nonspecific inflammatory infiltrate (Fig. 4).

## Discussion

The present study demonstrates that pars-solid GGN and pure GGN can have a different evolution during a 3-year follow-up. In patients with a history of cancer (or at high risk of lung malignancy), the presence of a pure or pars-solid GGN can be linked with new cancer or metastasis in a small percentage of cases (3/68 = 4,4%). However, if the pars-solid is present, FDG PET/CT is useful to identify the presence of lung malignancy with a sensitivity of 100%, while in case of pure GGN or small pars-solid GGN, CT imaging remains the preferred imaging modality.

During their follow-up period, cancer patients may develop numerous pulmonary abnormalities, from progression of disease with the appearance of lung metastases or a secondary lung neoplasm, to potential for disease and/or chemotherapy related immunosuppression. Chemotherapeutic drugs can also be pneumotoxic and cause lung parenchymal lesions. In a patient with known or suspected neoplasia, the diagnostic work-up for the appearance of one or more solid nodules on CT is well-defined since these have been proven to be metastases in 73% of cases (Seo et al., 2001). In contrast, the underlying cause of GGNs or part-solid GGNs is unclear. In the present study, we assessed the clinical significance of GGNs and part-solid GGNs in 68 oncology subjects during their observational period. All patients underwent serial CT scans for the characterization of nodules for a period of 3 years.

We found that only three patients showed a slow increase in the size of both ground-glass and solid components. Therefore, although the development of GGNs and part-solid GGNs in our case series is relatively rare (prevalence of 0,6%), a better interpretation of these findings is nevertheless important for clinical management. A few reports have described metastases with GGNs or part-solid GGNs components (Park et al., 2008; Attinà et al., 2013; Yanagitani et al., 2009). For example, Park and colleagues (Park et al., 2008) reported that the majority of part-solid GGNs were primary lung malignancies (67.8% adenocarcinoma and bronchiolo-alveolar carcinoma). These authors further showed that malignant lesions more likely had a larger ratio of solid to ground-glass component when compared to benign lesions (atypical adenomatous hyperplasia or AAH, focal fibrosis, and chronic granulomatous inflammation). In the recent report by Attina et al. (Attinà et al., 2013), the evolution of subsolid pulmonary nodules in cancer patients, seven out of 146 nodules were histologically confirmed as metastasis from extrapulmonary primary malignancy and five out of 146 confirmed as primary lung malignancy. Yanagitani et al. (Yanagitani et al., 2009) reported that ground glass metastases are uncommon findings in patients with previously diagnosed lung cancer and are difficult to distinguish from a second multifocal lung cancer. In their work, the authors reported that most of the GGNs in patients with pulmonary malignancy did not demonstrate any changes during follow-up and were histologically confirmed to be AAH. In contrast with Park’s series, in ours the progressive transformation of a GGN (9 mm) in a solid nodule (12 mm) with a surgical diagnosis of primary lung adenocarcinoma was present in only one patient. Moreover, we present some data also in contrast with Attinà et al. (Attinà et al., 2013), with a similar number of nodules tested (130 vs. 146) but with a different number of primary tumors. This difference can be due to the fact that most patients in our series (53/68, 78%) did not develop new lesions or changes in the original lesions. GGNs and part-solid GGNs remained unchanged both in the solid part (when present) and in the ground glass, for a median CT follow up of 18 months. This stability of the lesions would favor the non-metastatic hypothesis, even in the presence of metastasis in other organs. The lack of histological or biopsy information did not allow us to ascertain the origin of the nodules.

As reported in literature, the probability of malignancy is variable with regard to the densitometric features of the lesions (part-solid GGNs: 63%; GGNs:18%; solid PNs: 7%) (Leef & Klein, 2002). Guidelines for the management of GGNs and part-solid GGNs in patients without a previous diagnosis of cancer have been proposed and recently reviewed by Godoy and Naidich (Godoy & Naidich, 2009). In contrast with solid nodules guidelines, those for GGNs and part-solid GGNs do not distinguish patients at high risk from those at low risk for a pulmonary tumour. This is due to a: a) high incidence of adenocarcinomas (that frequently appear as GGNs or part-solid GGNs) in young nonsmokers; b) 2-year follow up not being long enough to characterize these lesions as benign or malignant, as they ought to be followed for at least 3 years. It should be noted that PET/CT does not have a role in the management of this kind of lesions since, as reported by many authors (Kim et al., 2012a; Chiu et al., 2012), adenocarcinomas of the lung show low FDG uptake and have low probability of nodal or distant metastasis (Kim et al., 2012b). In the present report, we found a significant uptake of FDG in only three patients with part-solid lesions that showed progressive changes at CT scans. FDG PET/CT cannot replace diagnostic CT in the definition of GGN due to: 1) the resolution power of a diagnostic CT is higher (5 mm vs 2 mm, respectively for PET/CT and diagnostic CT); 2) breathing during the scans (free breathing during PET/CT and deep-inspiration breath-hold during diagnostic CT) and 3) cost savings. For this latter point, based on the Italian tariffs, the costs of a thoracic diagnostic CT is significantly lower than a whole-body FDG PET/CT (77,67€ for CT without contrast enhancement-ce or 124,11€ with ce vs. 1.094,00 € for PET/CT, based on the Italian Healthcare System). However, PET/CT remains useful to characterize the pars-solid of the GGN, particularly in patients at high risk of metastases (i.e. patients with a history of cancer).

Conversely from GGNs, part-solid GGNs likely represent invasive malignancies with a high “a priori” probability and should receive a follow up CT at 3 months. If in this period, part-solid GGN has not disappeared or decreased in dimensions, it should be surgically resected after a FDG PET/CT examination. Controls that are too close in time are thus not indicated, and this is consistent with our findings, since nodules followed up at 1 month did not show any variation.

The actual guidelines for GGNs and part-solid GGNs (Naidich et al., 2013) are not be reproducible in subjects with other known neoplasms, even if the nodule characteristics are similar both in oncology and non-oncology subjects. From our analysis it emerged that the presence of stable and single or multiple GGNs in 46/53 patients (87%) during the follow-up of 48 months was indicative of a benign lesion, not an evolving one (such as AAH or small areas of fibrosis-AIS).

In many cases, oncology patients are immunodepressed due both to the neoplasm and to the specific therapy and therefore, they have a higher probability of infection. This latter condition can be masked by the general clinical conditions of the patient, particularly during pharmaceutical administration. In oncology subjects, among many of the pathogens that are correlated with opportunistic infections of the lung, some can determine parenchymal alterations such as GGNs or part-solid GGNs. In our series, GGNs rapidly disappeared (~2 months) in eight patients without any treatment. These lesions were correlated with small inflammatory processes, both non-specific or in relation to organizing pneumonia.

Moreover, many drugs can determine pulmonary toxicity, with the appearance in 10% of patients undergoing chemotherapy (Hurria et al., 2011). Bleomycine, cyclophosphamide, carmustine, busulfan and methotrexate are more frequently correlated with pulmonary toxicity, but more recent experimental drugs for oncology patients can cause similar alterations (Cooper Jr et al., 1986; Erasmus et al., 2002). Early diagnosis is important for the progressive worsening of the clinical condition, if the pharmacological agent is not withdrawn. In the present report, potentially pneumotoxic drugs (sorafenib, sunitinib, taxolo, cisplatino) were employed in nine enrolled patients. In four of these patients, CT examinations showed the appearance of multiple GGNs during therapy that disappeared after a median period of 3.5 months without any specific treatment.

Therefore in our series, all disappearing nodules during follow-up resulted pure ground glass at CT scan. Conversely, all increased nodules were part-solid GGNs. The radiological characteristics of lung nodules can be useful for oncologists in guiding the correct therapeutic choice during the follow-up of their patients.

Limitations of the present report are associated with the lack of bioptic or surgical confirmation in each case where pure and part-solid GGs did not demonstrate significant variations during follow-up. Moreover, being a retrospective study, follow-up periods were not homogeneous. Finally, the number of cases is limited, although it is not so different from other similar work that was conceived in a similar clinical-radiological setting. Lastly, we reconstructed CT images by using 2.0 and 2.5 mm in slice thickness. Although the current recommendations by Naidich et al. (Naidich et al., 2013) reported that to establish lesions as true GGNs, a thin CT section of 1 mm is preferable, whenever possible, other previous reports, such as Park et al. (Park et al., 2008), have demonstrated that a thickness between 1 and 5 mm can be considered. Moreover, the present study was conceived in an oncology setting where patients are frequently sent to multiple diagnostic examinations.

## Conclusions

In subjects treated with chemotherapy for primary tumor, lung nodules may be due to drug-induced inflammatory processes and not be related to the malignancy. The evolution of GGNs and part-solid GGNs may be affected by the side effects of chemotherapy and potential presence of inflammatory processes. Therefore, in accordance with International guidelines, a 3-year follow-up in these patients is justified by the fact that most of the nodules that eventually increased in size did so at a very slow growth rate. Finally, FDG-PET/CT is of limited use in excluding malignancy in this setting, but it shows high performance in cases of GGN with a solid component >5 mm.

## References

[CR1] JY O, Kwon SY, Yoon HI (2007). Clinical significance of a solitary ground-glass opacity (GGO) lesion of the lung detected by chest CT. Lung Cancer.

[CR2] Henschke CI, Yankelevitz DF, Mirtcheva R (2002). CT screening for lung cancer: frequency and significance of part-solid and nonsolid nodules. AJR.

[CR3] Austin JHM (2011). The incidental small pulmonary nodule and the Fleischner criteria 5 years later. Have we learned anything more?. J Thorac Imaging.

[CR4] Hasegawa M, Sone S, Takashima S (2000). Growth rate of small lung cancers detected on mass CT screening. Br J Radiol.

[CR5] Gaeta M, Volta S, Scribano E (1996). Air-space pattern in lung metastasis from adenocarcinoma of the GI tract. J Comput Assist Tomogr.

[CR6] Chun EJ, Lee HJ, Kang WJ (2009). Differentiation between malignancy and inflammation in pulmonary ground-glass nodules: the feasibility of integrated 18F-FDG PET/CT. Lung Cancer.

[CR7] Kim TJ, Park CM, Goo JM (2012). Is there a role for FDG PET in the management of lung cancer manifesting predominantly as ground glass opacity?. AJR.

[CR8] Chiu C-F, Liu Y-Y, Hsu W-H (2012). Shorter-time dual-phase FDG PET/CT in characterizing solid or ground-glass nodules based on surgical results. Clin Imaging.

[CR9] Godoy MCB, Naidich DP (2012). Overview and strategic management of subsolid pulmonary nodules. J Thorac Imaging.

[CR10] Seo JB, Im J-G, Goo JM (2001). Atypical pulmonary metastases: Spectrum of radiologic findings. Radiographics.

[CR11] Park CM, Goo JM, Kim TJ (2008). Pulmonary nodular ground-glass opacities in patients with extrapulmonary cancers: what is their clinical significance and how can we determine whether they are malignant or benign lesions?. Chest.

[CR12] Attinà D, Niro F, Stellino M (2013). Evolution of the subsolid pulmonary nodule: a retrospective study in patients with different neoplastic diseases in a nonscreening clinical context. Radiol Med.

[CR13] Yanagitani N, Kaira K, Ishizuka T (2009). Multiple lung metastases presenting as ground-glass opacities in a pulmonary adenocarcinoma: a case report. Cases Journal.

[CR14] Leef JL, Klein JS (2002). The solitary pulmonary nodule. Radiol Clin N Am.

[CR15] Godoy MC, Naidich DP (2009). Subsolid pulmonary nodules and the spectrum of peripheral adenocarcinomas of the lung: recommended interim guidelines for assessment and management. Radiology.

[CR16] Kim TJ, Park CM, Goo JM (2012). Is there a role for FDG PET in the management of lung cancer manifesting predominantly as ground glass opacity?. AJR.

[CR17] Naidich DP, Bankier AA, MacMahon H (2013). Reccomendations for the management of subsolid pulmonary nodules detected at CT: a statement from the Fleischner society. Radiology.

[CR18] Hurria A, Togawa K, Mohile SG (2011). Predicting chemotherapy toxicity in older adults with cancer: a prospective multicenter study. J Clin Oncol.

[CR19] Cooper JA, Merrill WW, Reynolds HY (1986). Cyclophospahmide modulation of bronchoalveolar cellular population and macrophage oxidative metabolism. Possible mechanisms of pulmonary pharmacotoxicity. Am Rev Respir Dis.

[CR20] Erasmus JJ, McAdams HP, Rossi SE (2002). Drug-induced lung injury. Semin Roentgenol.

